# Effect of alcohol on the central nervous system to develop neurological disorder: pathophysiological and lifestyle modulation can be potential therapeutic options for alcohol-induced neurotoxication

**DOI:** 10.3934/Neuroscience.2021021

**Published:** 2021-04-09

**Authors:** Zinia Pervin, Julia M Stephen

**Affiliations:** 1 Department of Biomedical Engineering, University of New Mexico, Albuquerque, NM 87131, USA; 2 The Mind Research Network and Lovelace Biomedical and Environmental Research Institute, Albuquerque, NM 87106, USA

**Keywords:** alcohol use disorder, central nervous system, oxidative stress response, neuropathology, blood-brain barrier dysfunction, neuroimaging, antioxidant

## Abstract

The central nervous system (CNS) is the major target for adverse effects of alcohol and extensively promotes the development of a significant number of neurological diseases such as stroke, brain tumor, multiple sclerosis (MS), Alzheimer's disease (AD), and amyotrophic lateral sclerosis (ALS). Excessive alcohol consumption causes severe neuro-immunological changes in the internal organs including irreversible brain injury and it also reacts with the defense mechanism of the blood-brain barrier (BBB) which in turn leads to changes in the configuration of the tight junction of endothelial cells and white matter thickness of the brain. Neuronal injury associated with malnutrition and oxidative stress-related BBB dysfunction may cause neuronal degeneration and demyelination in patients with alcohol use disorder (AUD); however, the underlying mechanism still remains unknown. To address this question, studies need to be performed on the contributing mechanisms of alcohol on pathological relationships of neurodegeneration that cause permanent neuronal damage. Moreover, alcohol-induced molecular changes of white matter with conduction disturbance in neurotransmission are a likely cause of myelin defect or axonal loss which correlates with cognitive dysfunctions in AUD. To extend our current knowledge in developing a neuroprotective environment, we need to explore the pathophysiology of ethanol (EtOH) metabolism and its effect on the CNS. Recent epidemiological studies and experimental animal research have revealed the association between excessive alcohol consumption and neurodegeneration. This review supports an interdisciplinary treatment protocol to protect the nervous system and to improve the cognitive outcomes of patients who suffer from alcohol-related neurodegeneration as well as clarify the pathological involvement of alcohol in causing other major neurological disorders.

## 1. Introduction

Alcohol is the most commonly used recreational beverage and drug of abuse among the adult population, alcohol-related death is the third leading preventable cause of death in the United States which accounts for more than 3.3 million global deaths annually [1],[2]. According to the 2018-National Survey on Drug Use and Health (NSDUH), 14.4 million people suffered from alcohol use disorder (AUD) in the US, and over 100,000 deaths were attributable to alcohol [3]. The World Health Organization reported that more than 200 health conditions including cancer, liver cirrhosis, and neurocognitive impairment were also attributed to alcohol consumption [2]. These chronic health conditions are progressive, cause a heavy economic burden to society, and decrease the quality of life for both patients and caregivers [4].

According to the National Institute of Alcohol Abuse and Alcoholism (NIAAA), AUD is defined as a chronic relapsing brain disease with an altered emotional state involving chronic alcohol abuse. This disorder may contribute to a considerable proportion of dementia, neurocognitive deficits, neuronal injury resulting from synaptic degeneration, nerve fiber demyelination, or blood-brain barrier dysfunction [5],[6]. In the presentation of the catastrophic or global loss of brain tissue, significant cortical-subcortical volume loss including white matter shrinkage occurs in patients with AUD, which is caused either by nutritional deficiency associated with alcoholic excitotoxicity or oxidative stress which results in alteration of various types of normal brain function [7],[8]. Furthermore, there is interest in the alcohol-induced metabolic disorder, Wernicke-Korsakoff syndrome (WKS), which is associated with thiamine deficiency and may contribute to severe neurological damage in the thalamus and hypothalamus [9],[10]. A combined effect of nutritional deficiency and ethanol toxicity may cause severe long-term effects and worsen the clinical manifestation of neurological impairment [11]. In general, persistent alcohol consumption may lead to gradual deterioration of psychological status with varying degrees of cognitive impairment including severe dementia [10]. Alcohol is the second leading cause of dementia (10%) among the adult population in the US after Alzheimer's disease (40–60%) [12]. The severity of neurological outcomes is associated in part with lifestyle factors including nutrition, amount, and term of alcohol consumption. Chronic alcoholic patients may develop severe malnutrition because they usually consume 50% of the calories from alcohol [13]. Alcohol consumption may have kindling effects and may increase epileptic episodes, cerebral infections, cerebrovascular lesions, and alter neurotransmitter systemic balance[14],[15]. Cognitive impairment may affect high order executive performance which may persist throughout the rest of life with secondary disabilities.

Neuron and myelin regeneration is a delicate process that requires different types of growth factors (nerve growth factor and brain-derived neurotrophic factor) to regulate and maintain neuronal homeostasis [16]. In AUD, essential growth factors of CNS homeostasis are downregulated by highly elevated alcohol metabolites acetaldehyde (AA) and reactive oxygen species (ROS), causing neuronal injury that leads to neurodegeneration [17]. Neurodegeneration, the opposite of regeneration, is when cells of the central nervous system stop working or die and usually perform actions more poorly with time in the presence of toxic or pathological conditions [18],[19]. Alcohol triggers abnormal protein accumulation, lysosomal dysfunction, and DNA damage which promotes neurodegeneration as well as accelerating the aging process of the brain [12],[4]. In contrast to AD and aging, alcohol's effect on the brain may be possible to slow, halt, or even reversible with alcohol abstinence because alcoholic brain shows shrinkage of brain tissue without significant loss of neurons, however, disrupted neuronal function or connection can be reestablished by modifying pathophysiology and lifestyle which can promote to maintain physiological homeostasis and cognitive function [20],[21].

Interestingly, previous research established the evidence of recovery and regeneration of cortical volume including white matter thickness in short-term abstinence as well as improvement in neurocognitive deficits particularly visuospatial abilities, working memory, and motor skills [22],[23]. The mechanism of alcohol-induced degeneration and alcohol abstinence regeneration is a complex phenomenon that is determined by a person's genetic characteristics, dominant brain activity, coexisting risk factors, and genetic process related to aging [24]. Sometimes, an immune-competent status with a pharmacological trigger or lifestyle modification can be a way to prevent the alcohol-induced neuronal insult and might play a significant role in brain recovery. This review will cover possible mechanisms of neurotoxicity in AUD to support an effort to establish a multidisciplinary therapeutic approach to prevent or reverse neurological damage.

## 2. Pathophysiology of alcohol metabolism and its consequence on BBB dysfunction

Despite thousands of published studies on alcohol-mediated neurological disturbance, the true mechanism of alcohol-induced cell death remains ambiguous. Many chronic AUD patients demonstrate neurocognitive and neurovascular injury associated with BBB dysfunction due to ethanol metabolites [25]. The BBB is a highly selective semipermeable membrane formed by brain microvascular endothelial cells (BMVEC). Pericytes and astrocytes connect the BMVEC assuring BBB structural tightness by binding with a tight junction which not only acts as a natural protector but also plays an important role to maintain normal brain homeostasis [26],[27]. Ethanol metabolites or neurotoxic substances may interact with the cytoskeletal structure of the brain to increase BBB permeability to start neuroinflammation [28],[29].

**Figure 1.**
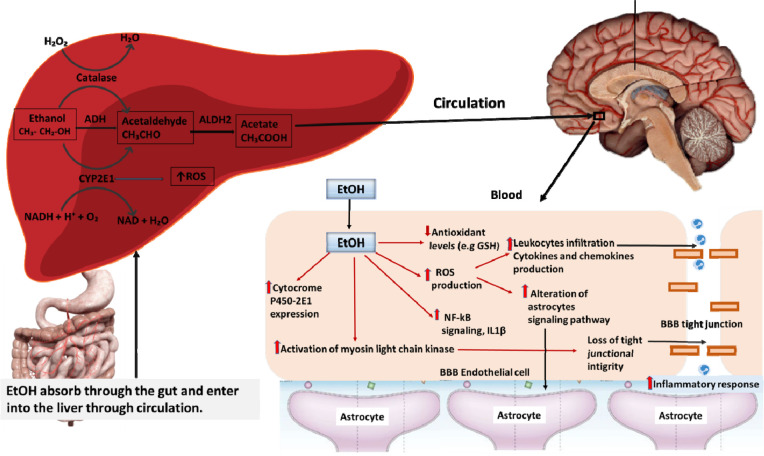
Schematic of ethanol metabolism through the liver and hypothetical involvement of ethanol metabolites for BBB dysfunction. In the presence of alcohol dehydrogenase (ADH) and cytochrome P450 enzymes, alcohol undergoes 1^st^ and 2^nd^ pass metabolism in the liver. Increased ROS and ethanol metabolites in the blood alter the signaling pathways of BBB endothelial cells and down-regulate the tight junction, which ultimately enhances leukocyte leakage and neuroinflammation [41],[42],[25].

Before discussing the effect of alcohol on BBB damage, we have to look through alcohol absorption and metabolism. The liver is the predominant organ for ethanol metabolism which usually occurs via two oxidative pathways mediated by alcohol dehydrogenase (ADH) and cytochrome P450 2E1 (CYP2E1) [30] (Figure 1). In brief, after drinking alcohol, absorption Occurs in the gastrointestinal tract then the liver converts the alcohol to acetaldehyde through the first-pass metabolism in the liver, this oxidation reaction is catalyzed by the alcohol dehydrogenase enzyme [31],[32]. After the first-pass metabolism, alcohol metabolites are distributed throughout the body, it goes to the brain through the blood vessels then it enters into the endothelial cells from the blood and alters the expression of signaling molecules which adhere to the BMVEC [33]. The metabolism of EtOH in the brain is controversial than the metabolism of acetaldehyde due to undetectable evidence of homogenous ADH activity in the whole brain. Animal experimental studies demonstrate the presence of cytochrome p4502E1 in the smooth endoplasmic reticulum of brain cells that are capable of Ethanol metabolism in brain by catalyzing the H_2_O_2_ with catalase enzyme [34]. However, the oxidation of acetaldehyde in brain cell is established because of ALDH (aldehyde dehydrogenase) have been well known to be found in mitochondria of brain cells [35]. ALDH converts acetaldehyde to acetate, acetate has further effects on brain including increase lipid peroxidation and free radicals production. EtOH exposure induces the catalytic expression of oxidative metabolizing enzymes which is parallel to enhancing the production of ROS (Figure 1).

It is known that during oxidative stress conditions the levels of oxidants are higher than the levels of antioxidants. So, ethanol indirectly decreases the antioxidant activity by increasing oxidative stress response. Alcohol-induced ROS production is believed to be specific to EtOH metabolism by cytochrome P450–2E1 (CYP2E1), which produces H_2_O_2_, superoxide, and free radicals. These free radicals, in turn, activate Rho kinase(ROCK/JNK) signaling to induce the release of vascular endothelial growth factor (VEGF) and inflammatory cytokines in brain endothelial cells (e.g. upregulation of ICAM-1and E-selectin, the release of IL-6) [36],[37].

Experimental studies on human brain tissue provide evidence of increased expression of CYP2E1 after chronic ethanol exposure and as a result of CYP2E1 mediated metabolism induces production of ROS and NO synthesis in the human brain [37],[38]. However, actions of EtOH metabolites depend on their concentration, ROS acts as active molecules at low concentration but at high concentration, oxidants convert as a transducer of the oxidative stress response and neurodegenerative agents [39]. As a consequence of exaggerated actions of ROS, transcription modulated lipid peroxidation is activated in neurons and increases the 4-HNE (lipid peroxidation products) level as well as decreases the neuronal cytoskeletal proteins [38],[40]. Disruption of neuron-specific neurofilaments or neuronal death initiates the primary process of alcohol-related neurodegeneration [37].

In the course of this phenomenon, further activation of astrocytes amplifies mitochondrial phosphorylation with downregulation of the tight junction which enhances the permeability of the BBB system. Thus, ethanol exposure results in BBB disruption by a complex immune-regulatory loop between BMECs and astrocytes. Evidence from animal models and cell culture reports further strengthens the idea that chronic excessive alcohol exposure downregulates the tight junction proteins (claudin, occludin, zonula occludens) which are responsible for maintaining BBB integrity [43]. Both acute and chronic alcohol exposure can increase the production of ROS and enhance peroxidation of lipids, protein, and phosphorylation of mitochondria resulting in decreased ATP production by disrupting phospholipid-containing cell membrane structure [44]. Astrocytes maintain the BBB integrity by forming paracrine interactions to coordinates the CNS blood flow and neural function between pericytes and CNS vasculature [45]. Alcohol-induced tight junction disassembly is usually mediated via activation of expression protein kinase C (PKC) which subsequently allows toxic substances to enter the brain which in turn affects CNS homeostasis. Incidental ablation occurs in astrocytes, pericytes vascular basement membrane results in unyielding leakage of leukocytes and immune complex molecules in and out of the brain, including secondary changes such as edema, inflammation, and hyper excitability may have appeared in white matter and cortical regions [32],[46] (Figure 1). Loss of astrocytes function to maintain the neurovascular coupling is not recovered by the proliferation of adjacent astrocytes resulting in long-term effect in neurovascular damage.

Empirical studies further show that ethanol-induced brain damage is mainly related to oxidative stress response from proinflammatory cytokines activated during alcohol intoxication. Proinflammatory cytokines NF-kB (transcription factor) mediate oxidative stress plays a role in the induction of anti-inflammatory and immune response signals, which appear to underlie neuronal degeneration and tissue atrophy [46],[47]. Cytokines are large families of secreted proteins that are transported from blood serum to neuronal tissue in response to oxidative stress-related alcohol neuroinflammation [47]. Increased cytokines particularly tumor necrotic factor (TNFα), interleukin IL-1β, and macrophage chemotactic protein 1 (CCL 2) expression cause neuroinflammation and insult of nerve axons in nociceptive synaptic terminals which leads to intracortical network miscommunication and neuropathy [48],[49].

As a result of BBB dysfunction, abnormal expression of water channel aquaporin (AQP) occurs which in turn causes cerebral edema by extravasating the water inside the brain tissue. The swelling of the brain plays a critical role in the pathogenesis of an extensive variety of CNS disorders including stroke, infection, and demyelination. Rodent brain studies provide evidence of the association of abnormal expression of water channel AQP4 in ROS-induced BBB dysfunction [50],[51]. The distribution and expression of AQP4 are important to maintaining CNS homeostasis. AQP4 is mainly arranged and organized in astrocytes and ependymal cells alongside myelinated fiber tracts [29],[52]. AQP4 may help astrocytes to maintain ion concentration by taking excess K^+^ inside the cell to activate the specific brain regions in exchange for rapid transfer of water out of the cell [52]. Alcohol-induced oxidative response interferes with the AQP4 activity and causes activity-related swelling of the extracellular space in white matter tracts (corpus callosum, optic chiasma, hippocampus, hypothalamic nuclei) where perinodal astrocytes fail to regulate the intracellular junctions at the nodes of Ranvier [52]. Inconsistent water movement in between CSF and brain parenchyma causes edema which appears to play a key role in the neurodegenerative process by facilitating a neuropathological environment. In the case of thiamine deficiency in chronic alcoholic abusers causes Wernicke korsakoff syndrome (WKS) due to its impaired metabolism of the mitochondrial oxidation to produce the brain energy and causes increase oxidative stress response and neuronal intoxication. Glucose serves as a primary fuel to mitigate the high demand for energy production in the central nervous system. The brain is highly vulnerable in a state of thiamine deficiency due to thiamine-dependent enzymes are required to glucose metabolism as well as mitochondrial ATP production for maintaining the CNS homeostasis, actions potentials, myelination and neuronal activity [53]. Impaired glucose metabolism decreases mitochondrial ATP production, thereby slow down the firing of the neuronal action potential, in addition, trigger lipid peroxidation, oxidative damage to CNS. Thus, Alcohol and its metabolites induce BBB disruption and neuroinflammation as well as alter the CNS homeostasis.

## 3. Impairment of glucose transport system leads to neurodegeneration

Previous research suggests a strong correlation between the impairment of glucose metabolism with subsequent neuronal loss at the interface of alcohol-induced BBB dysfunction which causes neurodegeneration in CNS [54],[55]. Therefore, disruption of BBB integrity may cause altered expression of the glucose transport channel protein (GLUT 1 and GLUT 3) and reduce uptake of glucose inside brain tissue [1]. About 90% of brain tissue depends on constant glucose supply as an energy source to maintain a dynamic function. GLUT 1 glucose transporter facilitates glucose transport from capillary endothelium to astrocytes then astrocytes metabolize some glucose molecules and transport these to neurons as fuel for anti-oxidation and tissue plasticity regeneration via the GLUT 3 transporter [56],[54]. In the preclinical period of neurodegeneration, glucose consumption is gradually diminished in neurons and glial cells of the hippocampus, corpus callosum, cerebral cortex, which induce lactate production, aerobic glycolysis, and structural plasticity in animal model [57],[55]. Eventually, clinical symptoms emerge and are associated with ataxia, spasticity, dementia, and mild to severe cognitive deficits. So, normal glucose homeostasis is important to maintain brain function; if any alteration or disruption occurs then it leads to neuronal toxicity with neuronal death results in neurodegenerative effect on cognitive function.

## 4. NMDA receptor-mediated neurotoxicity

N-methyl-D-aspartate (NMDA) is a primary excitatory brain neurotransmitter that binds to the glutamate receptor usually found in nerve cells. Depolarization and activation of the nerve action potential are maintained by the influx of different types of ions (Na^+^ and Ca^2+^) into the cell through the NMDA receptors [58]. Under normal circumstances, NMDA receptors play an important role in synaptic plasticity and signal transmission in the course of the cellular mechanism of learning, visuospatial memory, and buildup of working memory by neuronal synchronization through the intra-cortical communication of the central nervous system [59]. It is believed that alcohol acts as an antagonist for the NMDA receptor, so in the case of AUD, it causes hypofunction of the NMDA receptor which may result in neuronal network impairment with loss of synaptic plasticity [60]. To maintain normal neuronal function and homeostasis, the physiological actions of the NMDA receptor are required. Several controversial studies implicated that NMDA receptors are strongly involved with excitotoxicity which contributes to cell death and hamper the longevity of the cells [42],[58]. Recent evidence supports the hypothesis that excitotoxic events of NMDA receptors play a role in the formation of neurodegenerative diseases like Alzheimer's and Huntington's disease and affect normal brain function [11]. However, there is no established theory that delineates the use of alcohol as an NMDA receptor antagonist or medication for a neuroprotective role because successful implementation of NMDA antagonists would require blocking the excessive activation without interfering with the normal physiological function [61],[42]. In contrast, prior studies had shown that ethanol-induced blockage of the NMDA receptor could increase neurotoxicity by decreasing the expression of brain-derived neurotrophic factor (BDNF) during chronic alcohol administration [62]. Therefore, more studies are needed to establish the role of the NMDA receptor in the mechanism of neurodegeneration or neuro-regeneration in patients with AUD.

## 5. Astrocyte and oligodendrocyte associated neuronal dysfunction in AUD

Studies on the rodent and human brain delineated that excessive ethanol intake induces neuronal injury during various developmental stages including neurodegeneration and this type of ethanol-induced neurodegeneration seems to be connected with glial activation and neuroinflammation [23],[63],[64]. Astrocytes and oligodendrocytes play a crucial role in the molecular mechanism of signal conduction and neurotransmission in both gray and white matter. Besides, astrocytes, oligodendrocytes, and myelin protein take part in the maintenance of plasticity of gray and white matter [65]. In alcohol-related brain damage, ethanol and its metabolites have the potential to disrupt glial physiology and neurobiology in gray and white matter. Ethanol triggers the TLR4 receptor-dependent or -independent pathways of microglial activation which stimulates the NF-kB, interleukins IL1, IL6, CCL2, and in turn, evokes the expression of proinflammatory cytokines surrounding the astrocytes and oligodendrocytes [49],[64]. If this leads to an agglomeration of pro-inflammatory and neurotoxic mediators for a prolonged period in the glial environment, then it leads to neuroinflammation and neurodegeneration [63],[66]. In AUD, ethanol metabolites alter the expression of astrocytes and oligodendrocytes which leads to impaired cell to cell communication. Signal transmission and cell interaction are accomplished by the formation and maintenance of the myelin sheath which is usually disrupted by alcohol metabolites. Alcohol interferes with the neuronal homeostasis process including the ability to form colonies, integrate, differentiate, and mainly proliferate [11].

CNS inflammatory sequelae are believed to play a vital role in neuronal death as the pathway of neurodegeneration and inflammatory feedback is mainly mediated by microglial activation. In AUD, brain immune defense cells, microglia, are activate and express many proinflammatory genes including tumor necrotic factor α (TNF α), cyclo-oxygenase, NADPH enzymes which change the brain immune system and nerve cell functions [67],[68]. In the case of normal infection, this immunomodulation is limited and controlled by further immune signals but in AUD, chronic activation of microglia and sustained increases in microglial specific cellular markers activate inflammatory gene expression which may, in turn, cause neuronal death and disrupt the cellular integrity, ultimately leading to neurodegeneration. Therefore, a number of researchers believe that suppression of microglial activation could be a potential therapeutic to treat inflammation-mediated neurodegenerative disease [46].

## 6. Neuroimaging evidence of alcohol-induced neuroinflammation and neurodegeneration

Neuroimaging technology can observe the dynamic brain in a living body and allows researchers to conduct meticulous studies to gain insights into the effect of AUD on the human brain throughout the periods of chronic drinking, relapse, and abstinence. Brain images can be used to predict the severity of AUD by measuring the connectivity of neuronal features corresponding to the executive control network associated with different brain regions [69]. To explore treatment options for AUD, it is required to identify the status of neuronal injury and distinguish the reversible and irreversible neuronal loss with connectivity network in the course of alcohol intake from abstinence to heavy alcohol use. Structural MRI helps to visualize different cortical regions of the brain (gray, white matter, cortex, and midbrain) to examine the region-specific effects of chronic alcohol consumption [70]. Structural MRI findings in AUD provide evidence of mammillary body damage with hippocampal volume deficits that are also associated with the decreased axonal diameter in white matter, increased glial loss, or incorporation of newly formed astrocytes [71]. Structural MRI studies have also revealed the shrinkage of the frontal cortex, pons, and cerebellar hemispheres along with thinning of the corpus callosum as well as alcohol-related cortical abnormalities [72],[70]. These structural abnormalities give rise to the clinical symptoms of psychological impairment, dementia, amnesia, and motor dysfunction in patients with AUD [73]. Basal ganglia play an important role in regulating emotional and behavioral control but structural MRI images exhibit volume decreases of the hippocampus and basal ganglia in patients with AUD which may cause mental impairment with uncontrollable emotional aggression [74],[75].

In particular, MRI studies of individuals with AUD demonstrate widespread diffuse loss of both cortical white and gray matter thickness where disproportionate deficits of gray and white matter are more visible in older age compared to young patients [86]. The mechanism of neuronal damage and volume deficits in chronic drinking patterns that have been suggested is neuronal death with the destruction of glial structure which may be caused by the induction of pro-inflammatory cytokines and oxidative enzymes [87]. As a consequence of this damage, Wallerian degeneration and shrinkage of white matter occur in AUD which further leads to irreversible brain damage. Previous research provides evidence of neurogenesis in the adult brain as a process of pathological recovery, they have reported that the delicate process of neuro-generation occurs in the dentate gyrus of the hippocampus and persists into old age, after 65 years of age where the aging process usually halt the recovery process in the brain [88]–[90]. However, this physiological process can be interrupted by ethanol consumption before or after 65 years of age where ethanol metabolites hinder the growth of the progenitor's dendritic arbor to regulate the complexity of synaptic connections and thus may contribute to neurodegeneration [91],[92].

**Table 1.** Evidence-based study about the relationship between alcohol and neurodegeneration.

Neurodegenerative disorder	Study type	The number of subjects with alcohol exposure history. Cases/control	Brief Description of neurodegenerative risk.	References
Alzheimer's disease	Population-based longitudinal study	111/3,202	The increased risk, an excessive amount of alcohol enhances tau phosphorylation and β-amyloid accumulation in CNS.	[76], [77]
Parkinson's disease	NIH-AARP diet and health cohort study	1,113/306,895	Moderate risk, AUD activates cytochrome P450 2E1 and causes dopamine toxicity with the aggregation of α-synuclein in neuronal tissue.	[78], [79]
Amyotrophic lateral sclerosis (ALS)	Population-based case-control study	1557/2922	No influence, inconsistent risk.	[80], [81]
Generalized dementia	Ginkgo evaluation of memory study	512/3021	Considerable evidence, evidence of marked white matter disturbances, and alteration of glucose metabolism with decreasing neuronal density and volume decreases may be responsible factors for dementia in AUD	[82], [83]
Huntington's disease	Small study (42 subjects at johns-Hopkins hospitals)	***	Alcohol abuse has a strong effect on onset of motor symptoms in Huntington's disease, concurrent with depression syndromes.	[84], [85]
Multiple sclerosis	Population based cohort study	About 450/500000	Considerable evidence of elevated risk on concurrent alcohol abuse with cigarette smoking, heavy alcohol consumption may cause inflammatory demyelination and axonal degeneration.	[81], [84]

Note: *** no data available.

Multimodal imaging may be useful in predicting the cognitive outcomes and therapeutic success of substance use induced neurological disorder. The impacts of long term and short term alcohol use on cognitive functioning and neurodegeneration can be studied extensively by resting-state fMRI (functional magnetic resonance imaging) and task-based fMRI [69],[93]. Resting-state fMRI demonstrates the atypical dynamics in severe AUD [94] and task-based fMRI suggests altered neuronal network activity in executive control regions (Basal ganglia, SN) during task performance such as risk-taking, impulse control, and emotional oriented tasks [95],[96].

In general, structural MRI detects the proton of the hydrogen atom contained in fat and water of human body and reveals the tissue composition while diffusion tensor imaging (DTI) measures the diffusion of water protons in the brain tissue which demonstrates the integrity of white matter fiber tracts [97],[98]. Thus, the disruption of white matter microstructure has been extensively studied by DTI which reveals the axonal density, cytoskeletal structure, and myelin sheath characteristics along the long axis of projection fibers, which might be affected in AUD [99]. Ultimately DTI may be useful to examine BBB stability by detecting the anisotropic changes to quantify the permeability of water through the blood-brain barrier membrane [71]. DTI images bring evidence of osmotic demyelination which may be caused by the rapid osmotic fluid shift in the malnourished brain of individuals with AUD [75]. Central pontine and extra pontine myelinolysis is commonly seen in brain images of individuals with AUD with as assessed with DTI and occasionally represent different clinical manifestations [87]. Typical clinical symptoms of demyelination associated with AUD is the deterioration of mental status with seizures, dysarthria, paresis, sometimes linked to movement disorder such as catatonia, dystonia, and parkinsonism [100],[70]. Outcomes of brain damage are variable in patients with AUD and likely depend on the inflammatory or non-inflammatory loss of the myelin sheath. Eliminating alcohol from the diet may reverse the axonal damage with the regeneration of the myelin sheath in susceptible areas, but most cases of AUD brain damage cause inflammation-mediated demyelination which is contributed to irreversible damage like a diffuse axonal injury in AUD patients [101],[102]. In conclusion, alcohol-related irreversible brain damage in response to cerebral fluid shift mediated BBB dysfunction can be observed as a long term effect in WKS with diffusion tensor imaging [103].

Magnetic resonance spectroscopy (MRS) provides additional information about the molecular concentration and ethanol metabolites in the brain [104]. Proton-MRS can explore region-specific neurobiological status in combination with genetic mediated neurocognitive decline which has potential efficacy for future clinical management of AUD [105]. The largest MRS signals arise from N-acetyl aspartate (NAA), glutamate, glutamine, and choline-containing compounds (Cho) which are considered to measure neuronal integrity and normal brain function [106],[70]. MRS studies of the human brain have revealed a reduced level of NAA in several brain regions of patients with AUD which indicates neuronal injury. Similarly, studies in AUD patients have shown an elevated level of choline-containing compounds that usually provide evidence of demyelination but it is not consistent with alcohol withdrawal syndrome [71],[11]. According to earlier studies, alcohol withdrawal seizures commonly occur due to an imbalance between glutamatergic and GABAergic neurotransmission which can be detected by MRS of the human brain [107]. Proton-MRS can explore region-specific neurobiological status in combination with genetic mediated neurocognitive decline which has potential efficacy for future clinical management of AUD [105].

The main goal of neuroimaging techniques is to diagnose cognitive and functional abnormalities of the brain. To further capture these problems magnetoencephalography (MEG) with a prosaccade task can detect pathological alteration of neuronal activity in alcoholic patients compared to the normally developing healthy controls [108]. Schulte et al. demonstrated cognitive processing disturbance with neuronal desynchronization in adults with AUD in MEG study [100]. MEG data exhibits altered oscillatory neuronal activity and delayed evoked response in the alcoholic group that indicates the restricted ability to process somatosensory and multisensory response of high order cognitive performance during day-to-day interactions which may persist throughout life and ultimately leads to permanent cognitive impairment in chronic alcoholism [109],[110]. Accordingly, neuroimaging tools are required to observe the pathological changes and disease progression to figure out an applicable treatment agreement for AUD.

## 7. The potential therapeutic approach to prevent neurodegeneration

Despite the negative consequence of drinking alcohol, there is still hope for the recovery of alcohol-induced neurodegeneration. Neuro-regeneration (neuronal stem cell proliferation and formation of new neurons) generally depends on alcohol dosage, drinking duration, nutritional deficiency, stage of neuronal damage, and cellular components that correspond with cognitive functioning impairment. In AUD, alcohol alters the physiological status of the nervous system, may cause interruption of neuroprotective functions, and interfere with the absorption of certain nutrients which are necessary to maintain CNS homeostasis and brain cell development [111]. These factors may then result in loss of structure and function of multiple brain regions which induce alcoholic neurodegeneration [6]. Surprisingly alcohol abstinence could help individuals recover from the pathological state as well as improve cognitive function with sustained abstinence [67]. During abstinence, neural stem cells proliferate, differentiate, migrate and integrate into existing brain circuits to regenerate new neurons and re-establish the dendritic-axonal connection that contributes to learning [112],[67]. The longer the period of abstinence, the greater the chance of sustaining a healthy recovery of hippocampal dentate gyrus neurons, mammillary bodies, and return of executive functions including learning, memory, and other forms of cognition [75],[113]. Thus, abstinence regeneration is likely involved in blocking the pro-inflammatory gene expression and enhancing the high signaling cascades which contribute to the genesis of progenitor cells of neural stem cells, astrocytes, microglia, and oligodendrocytes in the course of trophic brain growth.

### 7.1. Reducing ROS by antioxidant and anti-inflammatory therapy

**Figure 2.**
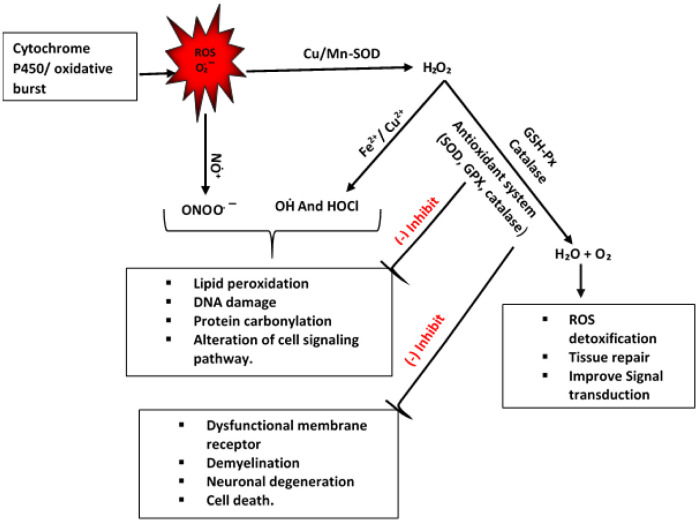
Alcohol-induced oxidative response which enhances the formation of certain free radicals (H_2_O_2_, OḢ, and HOCl), causes cell damage and neuronal degeneration. However, increased expression of the antioxidant system can inhibit the process of cellular dysfunction and trigger the tissue repair system.

The generation of ethanol metabolites and ROS related oxidative damage is believed to be induced by the pathogenesis of several neurodegenerative diseases such as Alzheimer's, Parkinson's, Huntington's diseases [25]. This oxidative damage is mainly mediated by O_2_·^−^, H_2_O_2_, and the highly reactive hydroxyl radical (HO·) which are byproducts of ethanol metabolism [114] (Figure 2). Therefore, maintaining the ROS level in the brain is required to regulate normal brain activity. Antioxidant activity is considered as enzymatic or non-enzymatic based on the mechanism of action involved to destroy the free radicals from the body [115]. The activity of antioxidant enzymes is significantly altered in the CNS of people who are chronically intoxicated with ethanol. There are some antioxidant enzymes mediated by protective signaling pathways such as superoxide dismutase (SOD) and heme oxygenase (HO^−1^) which can protect the neuronal tissue from oxidative damage by regulating transcription factor expression [116] (Figure 2).

Several transcription factors such as nuclear factor-erythroid 2 (NF-E2) related factor 2 (Nrf2), and peroxisome proliferator-activated receptor-coactivator (PGC-1) are responsible for upregulating the endogenous antioxidant enzyme systems to save the brain from alcohol-induced severe neuronal injury [113],[116]. Antioxidant enzyme heme oxygenase is thought to be highly associated with AD pathology and upregulated by the Nrf2 transcription factor. In the AD brain, GFAP positive astrocytes expressed heme oxygenase enzymes in the response to pharmacological activation of Nrf2 [117]. Overexpression of heme oxygenase can significantly decrease the intracellular cholesterol concentrations as well as a decrease in the exacerbations of amyloid-beta (Aβ) deposition in the neurodegenerative process of AD [118]. Also, heme oxygenase has another potential ability to cleave the tau protein by ubiquitin protease system which helps to inhibit the neurodegenerative process [119],[120]. Antioxidant SOD is regulated by PGC-1 factors and associated with human amyloid precursor protein (hAPP)-/Aβ-induced impairments in the AD brain [77]. Overexpression of SOD reduces the neurotoxicity by amyloid precursor protein and prevents memory deficits by reducing the activity of hippocampal superoxide. The most important exogenous antioxidants in the CNS are vitamin E, C, Omega 3 fatty acids, and selenium but both vitamin E and C levels in the CNS fall after alcohol exposure by ROS activity [121],[122].

We can trigger the antioxidant system by exogenous supplementation that can protect BBB from alcohol-induced toxicity. Some gases such as nitric oxide, CO, hydrogen sulfide known as medical gases can also be used as triggering factors of endogenous antioxidant enzymes (SOD) to scavenge the ROS from brain tissues [116]. Nitric oxide is a signaling molecule that is responsible to maintain physiological, immunological, and endothelial function also plays important role in inflammation as a precursor of ROS [123]. Under physiological limit, nitric oxide acts as a vasodilator and improves the oxygenation to the cells when tissue can defend themselves against oxidative damage through the antioxidant system. However, an imbalance presence of endogenous free radicals (NO) and antioxidants results in a pathological response of cells that contribute to the oxidative response. The anti-oxidative capacity of the CNS also depends on endogenous enzymatic antioxidant activity and exogenous antioxidants obtained by the organism through dietary intake. The endogenous enzymatic activity can be evoked by anaerobic exercise, depending on the antioxidant enzyme, changes of activity differ in time after the end of the anaerobic exercise [124]. Previous study findings revealed that an antioxidant named Butylated hydroxytoluene (BHT) precisely blocks elevation of alcohol-induced DNA binding of NF-κB, proinflammatory gene induction, and degradation of alcohol-induced DNA binding of cAMP-responsive element-binding protein (CREB) [125]. AUD may lead to thiamine deficiency or malnutrition because alcohol blocks the person's ability to absorb vitamins and nutrition's in the body [126]. Wernicke encephalopathy is a reversible condition in heavy alcohol use, usually caused by inadequate absorption or deficiency of thiamine that can be recovered by thiamine supplementation. However, Chronic thiamine deficiency in AUD leads to irreversible clinical condition known as Korsakoff syndrome that causes irreversible damage and are not improved with thiamine supplementation. Accordingly, rehabilitation treatment with vitamin supplementation (D, B6, A, C, thiamine, and B12) may help to improve the quality of life and halt the degenerative process. Stabilization of oxidative response and maintenance of physiological magnitude of endogenous antioxidant system play a key role to control the AUD associated neurotoxicity.

### 7.2. Pharmacological and lifestyle modifications

Currently, only five FDA-approved drugs are available to diminish the progression of neurodegenerative conditions. Four of them donepezil, rivastigmine, galantamine, tacrine, are based on acetylcholinesterase inhibition, and one of them, memantine, is an NMDA receptor antagonist [119]. Cognitive-behavioral therapy in conjunction with pharmacological options is developing interest as a treatment regime to enhance alcohol abstinence along with relapse prevention. The therapeutic agent, disulfiram was discovered for the treatment of alcohol dependence that blocks the conversion of acetaldehyde to acetic acid irreversibly results in accumulates the intermediate toxic product to develop an aversion to alcohol rather than proceed neurochemical actions of alcohol [127]. The adverse effect of disulfiram is outrageous over the clinical success towards preventing alcohol relapse. Anti-craving agents acamprosate and naltrexone are emerging concepts to control drinking. Naltrexone is an opioid receptor antagonist, found to be more effective to prevent relapse and maintain abstinence that reduces the rewarding effect of alcohol by generating fewer withdrawal effects [127],[128]. Acamprosate enhance the tolerance of alcohol withdrawal symptom by stabilizing the activity of N-methyl-D-aspartate (NMDA)-mediated glutamatergic excitation during early abstinence. However, their full clinical success has not been established and it depends on the administration, target, and severity of the disease.

Besides, immune therapy, N terminus-based antibodies immunization has a significant role in clearing the misfolded protein (Aβ and tau protein) but it is only effective at the earliest stage of disease [77]. Active Aβ immunotherapy (AN1792 vaccine trial) provided evidence of progression of mild to moderate dementia prior to death with a clearance of brain plaque in post-mortem follow-up [129],[130]. Tau Immunotherapy ACI-35 blocks the oligomerization of tau protein and improves motor activity, neurological deficiency with extended life expectancy in the tau transgenic rodent model [131],[132]. Therefore, novel therapeutic options are needed to treat the single or multi-target molecules of misfolded protein formation, oxidative stress damage, cognitive impairments, and synaptic integrity as well as the pro-inflammatory response in alcohol-induced neurodegeneration. Anti-inflammatory and neuroprotective agents can be one of the novel therapeutic options to treat or diminish the progression of neurodegenerative disease. Neuro-inflammation is activated by glial cells and produces inflammatory cytokines and toxic factors that cause neuronal death so if any anti-inflammatory drug controls the micro-glial activation and cytokine production then it may be another potential way to treat neurodegenerative disease [117],[17]. A case-control study on AUD suggests that baclofen has played an important role as a neuronal predictor in AUD; this study provides evidence of increased activation of the right anterior cingulate cortex and dorsolateral prefrontal cortex (which are involved in cognitive control) following baclofen treatment in comparison with the control group [133]. There is evidence of the success of neuroprotective agents over neurodegeneration because neuroprotective agents have the ability to activate neuronal restoration and survival pathways by correcting the oxidative damage or mitochondrial dysfunction, reducing microglial activation, and synaptogenesis or axonal genesis [134]. Among these factors, glial cell line-derived neurotrophic factor (GDNF) and mesencephalic astrocyte-derived neurotrophic factor (MANF) play a key role as neuroprotective agents in neuro restoration and neurogenesis to protect the neuron from oxidative damage [119],[112].

Interestingly, treatment options are not confined to pharmacology, conventional and clinical neurocognitive event-related potentials may have a significant role to improve the decision making, judgment, and social interaction ability which are impaired by addiction in heavy alcoholic individuals [135]. Delayed evoked response latency and decrease amplitude in ERP are commonly seen in AUD due to inhibitory mechanism and functional impairment associated with excessive alcohol consumption. Brain imaging (fMRI, MEG) cognitive ERP could be used to evoke the electrophysiological stimulus in the neuronal network to process the sensory, motor response to executing the high order event-related cognitive task [136]. Accordingly, cognitive ERP can help to dopamine release in cortical-striatal circuit results in hyper activation of the sensory-motor cortex over the alcoholic-cue inhibitory phenomenon. It is expected that this would have a constructive impact on the alcoholic individual's self-esteem, inducement, and compliance with the anticipated outcome. Transcranial magnetic stimulation (TMS) may be a relevant option to regenerate neurons and dendritic axon fibers by producing electric fields that spur action potential throughout the central and peripheral neurons [137]. TMS along with fMRI can be used to study neuronal connectivity and excitability, recently this treatment option was used to reduce dementia and induce remission for individuals with the major depressive disorder [138],[139]. Sometimes it is also experimentally used as a stimulant for myelination and to restore neuronal function in neurodegenerative diseases like multiple sclerosis and Alzheimer's disease [22],[140]. It is believed by several researchers that each glial cell has the ability to respond to the currents induced by TMS and play a role in enhancing the proliferation of adult progenitor and neuronal stem cells but there is less evidence on cell survival and cell differentiation process [66],[108]. Further research is needed to observe the direct effect of TMS on the blood-brain barrier and neuronal activity synchronization, which may introduce TMS as a complementary treatment option for AUD.

Lifestyle modification is also one of the most promising initiatives to reduce alcohol or age-related neurodegeneration as well as possible intervention strategies to control chronic disease or prevent the onset of dementia. Several lifestyle factors like aerobic and anaerobic exercise, an antioxidant-rich diet, limited alcohol consumption, neuropsychological therapy, and cognitive training have been demonstrated to improve cognitive function or postpone disease progression in AUD [141],[142]. The association between lifestyle modification and neurodegeneration in AUD is outlined in Table 2.

**Table 2.** The association between lifestyle modification and neurodegeneration in AUD.

Lifestyle and etiological factors	Risk assessment in AUD for developing neurodegeneration.	Protective strategy	References
Age	The brain is highly susceptible to induced neurodegeneration in old age (>65) with a history of chronic alcoholism.	Alcohol abstinence with antioxidants supplements can reduce the aging or degenerative process.	[4], [143], [82]
Genetic susceptibility	ApoE 4 genotype is a strong risk factor for developing AD. Moderate and heavy alcohol consumption during old age causes dementia with a major decline in learning ability among ApoE4 allele carriers.	Lower risk of developing dementia among ApoE 2 allele carriers.	[77], [144]
Smoking	Concurrent heavy smoking with alcohol drinking increases the incidence of dementia, AD.	Control drinking and smoking risk with vitamin A, C supplementation to decrease the risk of dementia	[145], [146]
Substance misuse	Cocaine use associated with AUD to facilitates neurodegeneration.	Stop drug use and add nutritional supplements	[147], [148]
Comorbid conditions	Cardiovascular disease, liver cirrhosis, stroke, traumatic brain injury can exaggerate the alcohol effects on the CNS.	Alcohol abstinence with treatment and control of the comorbid condition.	[149], [150]
Hypertension and hypercholesteremia	High blood pressure and high lipidemia have a relation with AUD to develop neurodegeneration in the elderly.	Reduce cholesterol and BP by controlling alcohol consumption	[149], [151]
Nutritional hypothesis	Alcohol interferes with vitamin absorption in the body and causes nutritional (thiamine, folate) deficiency which induces CNS degeneration	Choline, folate, Vitamin A, C, B1, B6 supplementation can postpone the alcohol-related degeneration.	[77], [152]
Physical exercise	Less physical activity enhances the chance to develop dementia in AUD	Aerobic and anaerobic exercise triggers the body's enzymatic antioxidants production and prevents neurodegeneration.	[124], [153]
Psychosocial status	Less education, depression, work complexity enhances neurotoxicity in AUD.	Increased mental activity and social networking, cognitive training, and education can help to prevent dementia.	[154], [149]

## 8. Conclusions

This review provides insight into alcohol mediated brain damage and establishes evidence that changes in the pathophysiology and lifestyle modifications can be an option for recovery and cell restoration in alcohol-induced neurodegeneration. Chronic alcohol abuse initially induces oxidative reduction response which leads to inflammatory activation with cytoskeletal destabilization of BBB integrity which further activates astrocytes to amplify the VEGF generation and increase the AQP4 expression, and thus finally causing BBB disruption and neuronal death. With the application of antioxidant therapy to control the oxidative response mediated inflammation, we expect to improve the outcome of neurocognitive function and structural stability of BBB with re-myelination and regrowth of neuronal processes to diminish neurodegeneration in patients with AUD. This review enhances the current state of knowledge regarding preventive approaches to alcohol-induced neurodegeneration by outlining the current understanding of alcohol-induced neurotoxicity while establishing possible therapeutic interventions to reduce further neurological impairment of patients with AUD.
